# Editorial: Genetic and Immunologic Response in Kawasaki Disease

**DOI:** 10.3389/fped.2022.876979

**Published:** 2022-04-12

**Authors:** Ho-Chang Kuo

**Affiliations:** ^1^Kawasaki Disease Center and Department of Pediatrics, Kaohsiung Chang Gung Memorial Hospital, Kaohsiung, Taiwan; ^2^College of Medicine, Chang Gung University, Taoyuan, Taiwan; ^3^Department of Respiratory Therapy, Kaohsiung Chang Gung Memorial Hospital, Chang Gung University College of Medicine, Kaohsiung, Taiwan

**Keywords:** Kawasaki disease (KD), genetic, immunologic response, coronary artery disease, intravenous immunoglobulin (IVIG)

Kawasaki Disease (KD) is a systemic vasculitis with unknown etiology until now. Immune, infectious, rheumatology or cardiologic diseases specialists or experts are all responsible for KD but not specific. KD affects mostly on children with age <5 years-old and intravenous immunoglobulin (IVIG) can provide good treatment response in the past two to three decades. Coronary artery lesions (CAL) are the main complication of KD and may have a life-long impact on children and/or families. Genetic or immunologic factors may play an essential role in the pathogenesis of KD.

There are more than 7,000 publications about KD from the PubMed database search, and most of them showed association with genes or immune response. This Research Topic enrolled articles of KD specific on the section of genetics and immune response from different nations or different areas that come from all over the world with the aim to get the immunopathogenic findings more clearly. The goal of this Research Topic is to provide more information about genetic or immunologic findings and even put them together to figure out the possibility of the etiology of KD from articles collected.

There were eight articles accepted for publication in this Research Topic including 64 authors, 3 original research, 1 brief research report, 1 mini review, 2 review, and 1 case report.

Buda et al. from Poland performed the first Genome-Wide Association Study (GWAS) in a population of Polish children and reported polymorphisms of genes KIF25, PTRPJ, SPECC1L, and RNP2 may be linked with the incidence of KD in Polish children according to 119 KD and 6,071 controls samples.

The result from this report may explain the incidence of KD in Poland; however geographic distribution of gene polymorphisms in Polish children is unknown.

Kuo et al. reported 24 healthy controls, 24 fever controls subjects and 49 KD subjects (with blood samples both before and after IVIG treatment) with total RNA from leukocytes and performed a quantitative polymerase chain reaction for the glycoprotein (HP, GRP84, and CLEC4D) genes in real time from Taiwan. The hyper-expression of these three genes was significantly associated with IVIG resistance, but not CAL formation.

Liu et al. reported a machine learning model to predict IVIG-resistant KD Patient from a retrospective study based on the Chongqing population of China. The study included 1,398 KD patients (with 158 cases of IVIG-resistance, 11.3%) hospitalized in 7 affiliated hospitals from January 2015 to August 2020. Eosinophilia is a major factor in a nomogram model and had high precision for predicting KD (1). Logistic regression nomograms, support vector machine (SVM), XGBoost and LightGBM prediction models were constructed and showed LightGBM prediction model for IVIG-resistant KD patients was superior to previous models. From literature review, there are few studies that showed comparison analysis between four kinds of machine learning model in KD. According to the results of logistic regression analysis, four independent risk factors were identified, including total bilirubin, procalcitonin, alanine aminotransferase, and platelet count.

Hsieh et al. from University of California San Diego reported that both children and adults possess an *Lactobacillus casei* cell wall extract (LCWE)-specific T cell repertoire that can be stimulated to express surface markers mediating homing to the vessels. Peripherally-induced regulatory T cells (iTreg) also responded to LCWE and potentially reverted to Th17. This finding showed compatible with previous report that Th17- and Treg-related cytokine and mRNA expression are associated with acute and resolving KD (2). Central memory T cells were also detectable and were more abundant in adults. The potential homing to the vessels of LCWE-specific T cells was suggested by the expression of CCR6 and CD31. However, there is currently no evidence that these cells actually participate in vascular inflammation in humans.

Sharma et al. reviewed the epigenetics including methylation, micro-RNAs, and long non-coding RNAs in KD and discuss how these mechanisms can help us better understand the disease pathogenesis and advance the development of new biomarkers.

Chang et al. reviewed the immunopathogenesis and immunotherapies for KD including aspect of infection, autoimmunity, immunopathogenesis, immunogenetics and clinical phenotypes, ethnical differences and genetic susceptibility, epigenetic factors in the immunoregulation, evolvement of immunotherapies and perspective of immunotherapies for KD with IVIG-resistance. The review provide more detail clinical information for KD from immunopathogenesis and immunotherapies.

Brar et al. reviewed the impact of electronic cigarettes (e-cigarettes or vaping) on the pandemic of SARS-CoV-2 and multisystem inflammatory syndrome (MIS-C). Coronavirus disease-19 (COVID-19) in children is usually mild but some are susceptible to a KD-like multisystem inflammatory syndrome in children (MIS-C) in the convalescent stage (3). The SARS-CoV-2 virus targets angiotensin converting receptor (ACE receptor), and studies also showed nicotine-based e-cigarettes or vaping cause oxidative stress and resulting in the upregulation of ACE2, which might worsen acute respiratory distress syndrome (ARDS) in MIS-C. The mini-review highlighted that adolescents using e-cigarette will affect pulmonary defenses against SARS-CoV-2 through upregulation of the ACE2 receptors (primary target of SARS-CoV-2).

Takasago et al. reported comprehensive temporal analyses of serum cytokine kinetics for the entire course of disease, along with an investigation of clinical symptoms, in a Japanese patient with MIS-C and KD. The findings regarding cytokines that changed during the course of disease may provide useful information for elucidating disease status and selecting therapy.

Taking together, in this Research Topic, GWAS, epigenetic modification, glycoprotein, serum kinetic cytokines change, and LCWE-specific T cell were reported to make the genetic and immune response of KD comprehensible. Machine learning model to predict IVIG-resistant in KD, e-cigarette effect and immunopathogenesis as well as immunotherapies review provided useful information for elucidating disease status and selecting therapy in KD and even MIS-C.

## Author Contributions

H-CK prepared and proofed the manuscript.

## Conflict of Interest

The author declares that the research was conducted in the absence of any commercial or financial relationships that could be construed as a potential conflict of interest.

## Publisher's Note

All claims expressed in this article are solely those of the authors and do not necessarily represent those of their affiliated organizations, or those of the publisher, the editors and the reviewers. Any product that may be evaluated in this article, or claim that may be made by its manufacturer, is not guaranteed or endorsed by the publisher.

## References

[1] Liu X-P Huang Y-S Xia H-B Sun Y Lang X-L Li Q-Z. A nomogram model identifies eosinophilic frequencies to powerfully discriminate Kawasaki disease from febrile infections. Front Pediatr. (2020) 8:559389. 10.3389/fped.2020.559389 33363059 PMC7759494

[2] Guo MH Tseng WN Ko CH Pan HM Hsieh KS Kuo HC. Th17-and Treg-related cytokine and mRNA expression are associated with acute and resolving Kawasaki disease. Allergy. (2015) 70:310–8. 10.1111/all.12558 25585854

[3] Chen MR Kuo HC Lee YJ Chi H Li SC Lee HC. Phenotype, susceptibility, autoimmunity, and immunotherapy between kawasaki disease and coronavirus disease-19 associated multisystem inflammatory syndrome in children. Front Immunol. (2021) 12:632890. 10.3389/fimmu.2021.632890 33732254 PMC7959769

